# The Simple One-Step (SOS) Stool Processing Method for Use with the Xpert MTB/RIF Assay for a Child-Friendly Diagnosis of Tuberculosis Closer to the Point of Care

**DOI:** 10.1128/JCM.00406-21

**Published:** 2021-07-19

**Authors:** Petra de Haas, Bazezew Yenew, Endale Mengesha, Andrii Slyzkyi, Zewdu Gashu, Manon Lounnas, Ephrem Tesfaye, Ahmed Bedru, Edine Tiemersma, Kristin Kremer, Misikir Amare, Getu Diriba, Betselot Zerihun, Tilaye Gudina, Ben Tegegn, Maryline Bonnet, Challa Negeri, Eveline Klinkenberg

**Affiliations:** a KNCV Tuberculosis Foundation, The Hague, The Netherlands; b Ethiopian Public Health Institute, Addis Ababa, Ethiopia; c KNCV Tuberculosis Foundation Ethiopia Office, Addis Ababa, Ethiopia; d UMR MIVEGEC IRD-CNRS-University of Montpellier, IRD, Montpellier, France; e National Tuberculosis and Leprosy Control Program, Addis Ababa, Ethiopia; f Addis Ababa Regional Health Bureau, Addis Ababa, Ethiopia; g IRD UMI 233 Trans-VIHMI-UM-INSERM U1175, Montpellier, France; h Department of Global Health and Amsterdam Institute for Global Health and Development, Amsterdam University Medical Center, Amsterdam, The Netherlands; University of Manitoba

**Keywords:** tuberculosis, children, stool, diagnosis, Ethiopia, simple one-step stool method, Xpert MTB/RIF, bacteriologically confirmed, point of care, sputum

## Abstract

Young children cannot easily produce sputum for diagnosis of pulmonary tuberculosis (TB). Alternatively, Mycobacterium tuberculosis complex bacilli can be detected in stool by using the Xpert MTB/RIF (Ultra) assay (Xpert). Published stool processing methods contain somewhat complex procedures and require additional supplies. The aim of this study was to develop a simple one-step (SOS) stool processing method based on gravity sedimentation only, similar to Xpert testing of sputum samples, for the detection of M. tuberculosis in stool samples. We first assessed whether the SOS stool method could provide valid Xpert results without the need for bead-beating, dilution, and filtration steps. We concluded that this was the case, and we then validated the SOS stool method by testing spiked stool samples. By using the SOS stool method, 27 of the 29 spiked samples gave valid Xpert results, and M. tuberculosis was recovered from all 27 samples. The proof of principle of the SOS stool method was demonstrated in routine settings in Addis Ababa, Ethiopia. Nine of 123 children with presumptive TB had M. tuberculosis-positive results for nasogastric aspiration (NGA) samples, and 7 (77.8%) of those children also had M. tuberculosis-positive Xpert results for stool samples. Additionally, M. tuberculosis was detected in the stool samples but not the NGA samples from 2 children. The SOS stool processing method makes use of the standard Xpert assay kit, without the need for additional supplies or equipment. The method can potentially be rolled out to any Xpert site, bringing a bacteriologically confirmed diagnosis of TB in children closer to the point of care.

## INTRODUCTION

The diagnosis of pulmonary tuberculosis (PTB) in young children remains challenging, especially at the primary health care level, where sick children usually present first (1, 2). Children with PTB often present nonspecifically, without typical signs and symptoms of tuberculosis (TB), especially HIV-positive or malnourished children. Moreover, a microbiological diagnosis often cannot be obtained because young children cannot produce a sputum specimen on command and they often swallow their sputum when they cough. In such cases, a sputum sample can be obtained only through nasogastric aspiration (NGA) or sputum induction. These procedures are fairly invasive and poorly accepted and require specialized equipment and skills, which often are lacking at the lowest levels of health care. Because TB in young children is often paucibacillary, diagnostic methods with high sensitivity are needed to maximize the likelihood of detection of Mycobacterium tuberculosis bacilli. A microbiological diagnosis reduces the risk of misdiagnosis, especially for drug-resistant TB, and the risk of delayed initiation of effective treatment (3).

A sensitive diagnostic method that uses noninvasively collected specimens that can easily be obtained, processed, and tested at or near the point of care would increase access to a confirmed diagnosis, which is currently strikingly low, even in hospitals (4). Recently, the World Health Organization (WHO) recommended stool for the diagnosis of TB in children (5). Stool can easily be collected, and various recent studies have demonstrated that the molecular cartridge-based Xpert MTB/RIF assay (Cepheid, Sunnyvale, CA, US) or the more sensitive Xpert MTB/RIF Ultra assay can be used to detect M. tuberculosis in stool (6–8). Moreover, GeneXpert instruments are widely available at the secondary care level, and availability at the primary care level is rapidly expanding in most countries, with the aim to reach the End TB strategy (9). Thus, the application of Xpert testing with stool provides the opportunity for the sensitive and noninvasive diagnostic workup for the diagnosis of TB in children near the point of care.

Although stool collection and the Xpert assay are relatively easy procedures, the currently available procedures to process the stool before it can be tested in the Xpert assay are not. Most published stool processing methods for the Xpert assay are complex, labor-intensive, and time-consuming, include centrifugation, and need well-equipped infrastructure (7, 10, 11). More simple centrifuge-free methods include the method developed by Banada and coworkers (12, 13) and the optimized sucrose flotation (OSF) method developed by the TB-Speed consortium (14). However, these centrifuge-free methods still require additional specimen-processing steps, including the preparation of a buffer. An even simpler two-step (TS) method, which requires only a tube containing phosphate-buffered saline (PBS) in addition to the materials provided with the Xpert cartridge, was applied by Andriyoko and colleagues (15). Moreover, all of these methods require specific biosafety measures due to specimen manipulation before the M. tuberculosis bacilli are inactivated. The aforementioned disadvantages of the currently available stool processing methods limit the widespread application of the Xpert assay for the detection of M. tuberculosis in stool at the primary health care level.

We hypothesized that stool processing for use in the Xpert assay could be as simple as sputum processing, with simple addition of the stool directly to the sample reagent (SR) (provided in the Xpert kit) to release the bacteria from the feces and to inactivate the M. tuberculosis bacilli, followed by sedimentation by gravity, assuming that the M. tuberculosis bacilli would float to the top in the watery solution due to their lipid-containing cell wall. The stool processing method would then be reduced to only one release/sedimentation step; therefore, we refer to it as the “simple one-step (SOS) stool method.” This paper describes the three phases that led to the development of the SOS stool method, i.e., two series of laboratory experiments, followed by a proof-of-principle study in which the SOS stool method was applied to the stool of children with presumptive PTB in a routine setting in Addis Ababa, Ethiopia.

## MATERIALS AND METHODS

### Study design and clinical samples.

This study consisted of three phases. In the first phase, the SOS method for the detection of TB in stool was developed by testing our hypothesis that stool processing for the GeneXpert system could be as simple as sputum processing. In this phase, we conducted a series of experiments to assess whether the SOS stool method would yield successful Xpert assay results or whether additional steps, such as dilution (15), bead beating, or filtration (12), would be needed to optimize the PCR. The experiments for the method development were conducted at the National Tuberculosis Reference Laboratory (NTRL) at the Ethiopian Public Health Institute (EPHI) in Addis Ababa, Ethiopia, in June 2017 by using stool obtained from two healthy child volunteers.

In the second phase, the SOS stool method was validated by comparing the results of this method with those of a slightly adapted TS method (15) in testing stool samples spiked with M. tuberculosis bacteria. For this method validation, anonymized stool samples were obtained from adults (≥18 years) who were hospitalized at the University Teaching Hospital of Montpellier in France. These experiments were conducted at the University Teaching Hospital TB laboratory in Montpellier, France, in April 2018.

In the last phase, the proof of principle for the SOS stool method was investigated. For this part of the study, stool samples were collected from children ≤10 years of age who were enrolled in an ongoing study conducted at selected health facilities in Addis Ababa, Ethiopia. In this study, children presenting with presumptive TB were subjected to routine clinical examination. All consecutively enrolled children for whom routine NGA was requested for Xpert testing were asked to also submit a stool sample. Enrollment started in December 2018; for the analysis in this paper, data from children enrolled through December 2019, when TB was detected in the NGA samples from 10 children, were included. This study, part of the NoPain4kids project, was approved by the EPHI institutional review board (protocol EPHI-IRB-134-2018). Written informed parental consent was obtained from the parents/legal guardians for each child enrolled. Data confidentiality was ensured, and participant names or any other personal identifiers were not included in the data set for analysis and were used only to link laboratory results to children for caretaking when needed, following routine surveillance guidelines for medical records. All children diagnosed with TB according to clinicians’ decisions were treated in accordance with national guidelines.

### Method development (phase 1).

Various amounts (0.3 g to 1.0 g) of two stool samples with a soft consistency were used to test different stool processing protocols before testing with the Xpert MTB/RIF assay (Cepheid) for the detection of M. tuberculosis and resistance to rifampin. This testing was done on a GeneXpert instrument (Cepheid), following the instructions of the manufacturer. The protocol for the proposed SOS stool method consisted of the standard protocol for sputum processing for Xpert testing (16, 17) with a few minor modifications (see details below), i.e., a single release/sedimentation step. In addition, various other stool processing protocols were tested, with the following additional steps: (i) addition of glass beads to improve sample homogenization and potentially increase the release of M. tuberculosis, following the method described by Banada et al. (12); (ii) dilution with PBS, resembling the TS method described by Andriyoko et al. (15); and (iii) filtration following the method described by Banada et al. (12). Also, protocols that consisted of various combinations of these additional steps were evaluated. Figure S1 in the supplemental material shows a schematic overview of the series of laboratory experiments and more details on how these were performed.

### Method validation (phase 2).

The SOS stool processing method developed in phase 1 of this study and a slightly adapted TS stool processing method were validated by testing spiked stool samples using the Xpert MTB/RIF Ultra assay (Cepheid) in the GeneXpert instrument (Cepheid), following the instructions of the manufacturer. Of the total of 15 different stool samples that were used to create spiked samples, 9 had a soft consistency, 2 were semisolid/solid, and 4 were semiliquid/liquid. Fourteen of these stool samples were divided in four aliquots of 1 g, and one stool sample was divided into two aliquots of 1 g, yielding a total of 58 samples and 29 pairs of stool samples to be processed by each of the two methods. Each aliquot was spiked by adding 10 or 100 μl of prequantified M. tuberculosis cell stocks, resulting in a final concentration of approximately 10^3^ M. tuberculosis CFU/g, as described by Lounnas et al. (14).

The SOS stool processing procedure was conducted following the standard protocol for sputum processing for Xpert testing (16, 17) with a few minor modifications with regard to the volume of SR used, the duration of the last incubation step, and how to handle the SR-stool mixture when transferring the sample into the cartridge. In short, a portion of 0.8 to 1.0 g stool was picked, weighed, and added directly into the 8 ml SR in the SR bottle of the Xpert kit (Cepheid). The SR-stool mixture was shaken for 30 s, left at room temperature for 10 min, shaken vigorously again for 30 s, and left at room temperature for another 10 min for cell debris to settle by gravity sedimentation. Then, without moving the SR bottle and without disturbing the sediment, 2 ml of the upper layer of the “debris-free” supernatant was carefully transferred into the Xpert cartridge. The cartridge was then transferred into the GeneXpert instrument and processed following the instructions of the manufacturer.

**FIG 1:**
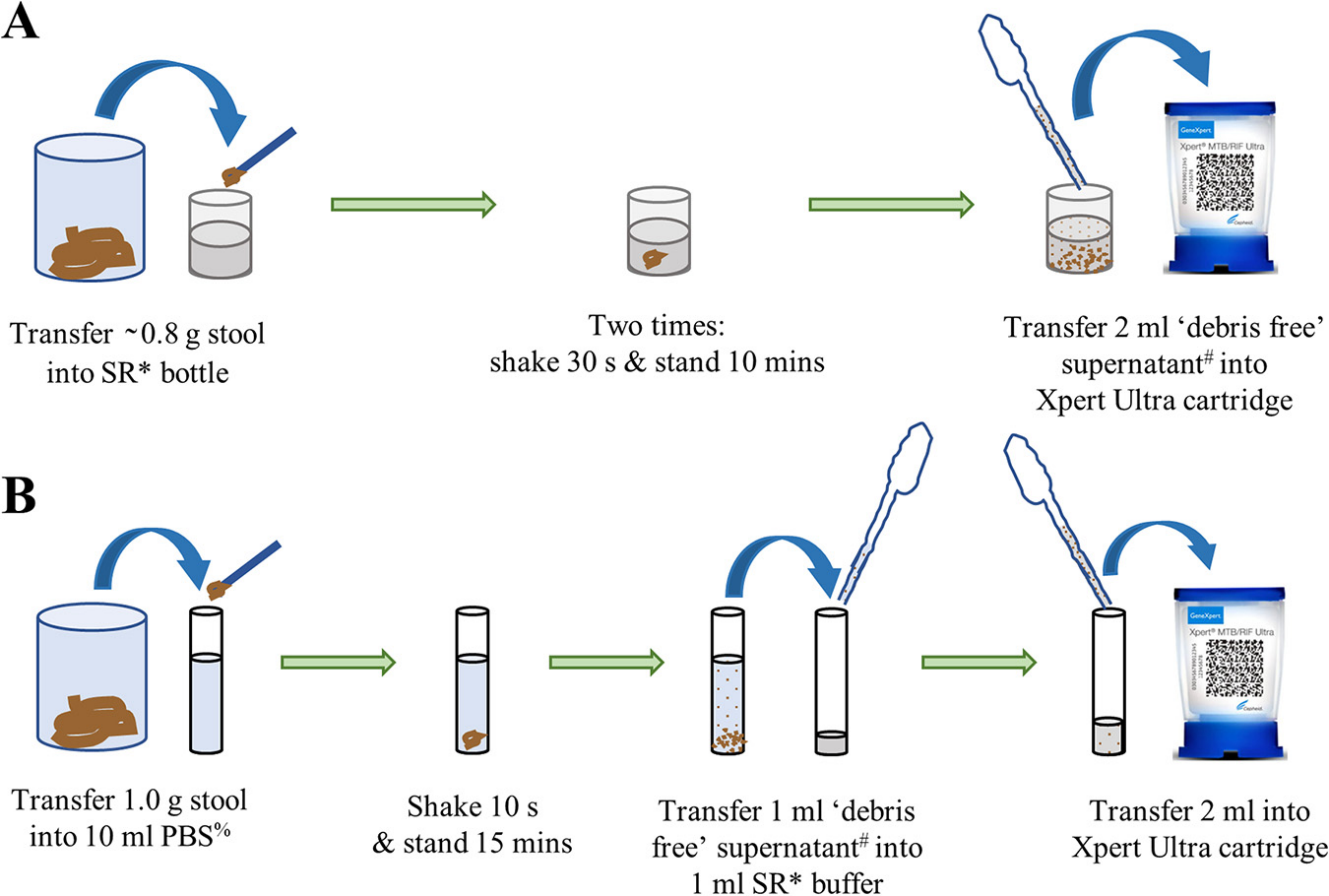
Schematic overview of the two methods that were used in the validation phase of this study, in which spiked stool samples were tested for the presence of M. tuberculosis by using the GeneXpert Ultra system. (A) SOS stool processing method. (B) Slightly adapted TS stool processing method described previously by Andriyoko et al. (15). *, The SR (Cepheid) is an 8-ml mixture of sodium hydroxide (pH of >12.5) and isopropanol provided with every Xpert Ultra cartridge. #, After gravity sedimentation of the organic debris, carefully, without lifting the bottle and without disturbing the sediment, 2 ml of the upper layer of the debris-free supernatant was transferred. %, The PBS is pH 7.4.

The TS method described by Andriyoko et al. (15) was used with only a difference in the volume of SR used; 1 ml supernatant was added to 1 ml SR instead of 2 ml. Figure 1 shows a schematic overview of both the SOS method and the TS method.

### Proof-of-principle study (phase 3).

For the proof-of-principle study, the NGA specimen from each child was split into two samples during collection. One NGA sample was processed the same day, on site, following the standard protocol for sputum processing for Xpert testing (16, 17) by using the Xpert MTB/RIF Ultra assay. The second NGA sample was transported to the NTRL at EPHI and then processed within 24 h by using the NaOH/*N*-acetyl-l-cysteine (NALC) method to perform solid (Lowenstein-Jensen [LJ] medium) and liquid (mycobacteria growth indicator tube [MGIT]) cultures (18).

Stool was processed and tested by using the SOS stool processing method described above in phase 2 and summarized in Fig. 1, with the only difference being that the stool was not weighed. Laboratory staff members were trained and provided with instructions on how to visually pick ∼0.8 g of stool.

### Data collection and analysis.

Semiquantitative Xpert results and the cycle threshold (*C_T_*) values for the sample processing control (SPC) were captured directly from the GeneXpert instrument. We used the semiquantitative Xpert results following the manufacturer’s categories, which represented the M. tuberculosis bacterial load detected in the sample (17), as follows: M. tuberculosis detected, high (*C_T_* value for the *rpoB* gene of ≤16); M. tuberculosis detected, medium (*C_T_* value for the *rpoB* gene of 16 to ≤22); M. tuberculosis detected, low (*C_T_* value for the *rpoB* gene of 22 to ≤28); or M. tuberculosis detected, very low (*C_T_* value for the *rpoB* gene of >28). The Xpert Ultra results contained one additional category, namely, M. tuberculosis trace detected (*rpoB* gene not detected but IS*6110*/IS*1081* detected).

For comparison of the various protocols during the method development, we calculated the difference (Δ*C_T_*) between each test’s SPC *C_T_* value and the reference SPC *C_T_* value (obtained by testing undiluted SR). The M. tuberculosis recovery rate of testing spiked samples was defined as the proportion of samples with M. tuberculosis detected among all spiked samples. The M. tuberculosis detection rate of testing spiked samples was defined as the proportion of samples with M. tuberculosis detected among all samples tested with a valid Xpert Ultra test result. We applied the following statistical methods to compare the results of paired samples tested with the TS method and the SOS method: the difference in bacterial loads between the two methods was tested using the Wilcoxon matched-pairs signed-rank test, while the difference in mean SPC *C_T_* values was tested using a paired *t* test. In the proof-of-principle study, standardized forms were used to capture information on participants, laboratory tests, diagnosis, and TB treatment. Data were entered into Epi Info version 3.5.4 (Centers for Disease Control and Prevention). Analysis was conducted using Stata version 15.1 (StataCorp LLC, USA). The McNemar test was used to test for differences in proportions of unsuccessful results for Xpert assays with NGA samples versus stool samples, while the differences in mean SPC *C_T_* values were tested using the paired *t* test. Results were statistically significant if the *P* value was <0.05.

## RESULTS

### Method development.

Eighteen experiments in which different stool processing protocols were performed with various amounts of stool obtained from healthy children were conducted (see Fig. S1 and Table S1 in the supplemental material). Overall, the SPC *C_T_* values increased with the quantity of stool used for testing, but we concluded that 0.8 to 1.0 g of stool generally yielded successful Xpert test results with the SOS stool method. The SOS stool method showed slightly lower SPC *C_T_* values than when bead-beating and/or dilution steps were added to the stool processing protocol, suggesting that the SOS method without the addition of these steps may be slightly more sensitive. Protocols including filtration, a relatively complex step, yielded lower Δ*C_T_* values than protocols without filtration (see Table S1). Based on the first series of experiments, we concluded that the SOS stool method produced valid test results.

### Method validation.

**FIG 2:**
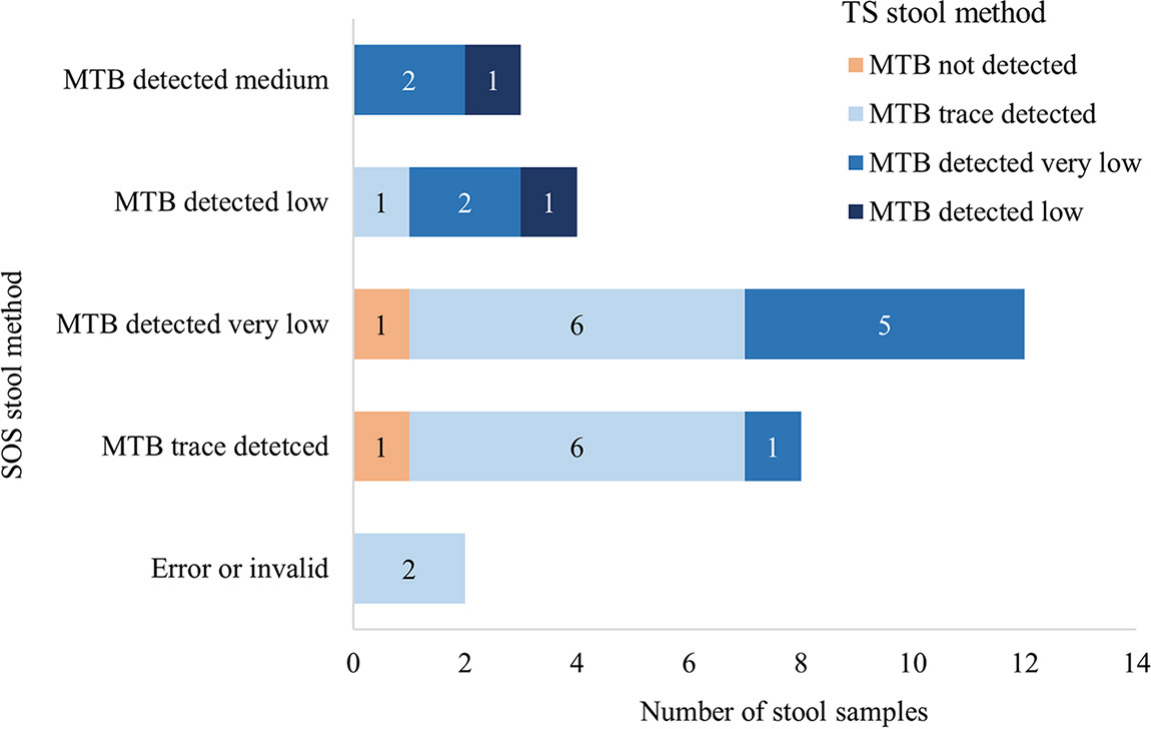
Xpert Ultra assay results from testing of 29 spiked stool samples processed by using the SOS stool method and the TS stool method, according to the semiquantitative Xpert Ultra results. For example, the SOS method had 8 samples with M. tuberculosis (MTB) trace detected, while the results of the TS method for these 8 samples included 1 M. tuberculosis not detected, 6 M. tuberculosis trace detected, and 1 M. tuberculosis detected, very low.

Twenty-nine pairs of spiked stool samples were tested by both the SOS stool method and the TS stool method, as a proxy for the ability of these methods to detect M. tuberculosis bacteria. With the TS method, all 29 of 29 spiked samples gave valid results, while this method failed to detect M. tuberculosis in 2 samples. With the SOS stool method, 1 sample gave an invalid result and 1 demonstrated “error code 2008,” thus yielding 27/29 valid results. M. tuberculosis was detected in all of those 27 samples. Thus, the M. tuberculosis recovery rate was 93.1% (95% confidence interval [CI], 77.2 to 99.2%) (27/29) for both methods, and the M. tuberculosis detection rate was 100.0% (27/27) for the SOS stool method and 93.1% (27/29) for the TS method. Figure 2 compares the semiquantitative Xpert results for the SOS and TS methods. Overall, the SOS stool method resulted in a higher level of detection, represented by detection of a statistically significantly higher bacterial load, than the TS method (*P* < 0.001). Among all 27 spiked stool sample pairs that gave valid results with both methods, the bacterial load measured with the SOS method was higher than that measured with the TS method for 14 pairs (51.9%), while it was the other way around for only 1 pair (3.7%); for the remaining 12 pairs (44.4%), the detected bacterial loads were equal (Fig. 2). Samples processed by the TS method are more dilute, compared to those processed by the SOS method, due to the extra step of adding PBS buffer. In a comparison of the 13 pairs that had SPC *C_T_* values available for both methods, the SOS method gave slightly higher SPC *C_T_* values than did the TS method (mean SPC *C_T_* values of 33.2 ± 3.3 versus 28.7 ± 2.8; *P* = 0.003). Further details on the 29 pairs of spiked samples can be found in Table S2.

### Proof-of-principle study.

Among 147 consecutively enrolled children for whom NGA specimens were collected, 10 had M. tuberculosis-positive NGA samples. Of these, 123 children (83.7%) also had a stool specimen collected. Among these 123 children, 9 (7.3%) had NGA samples that were M. tuberculosis positive by Xpert (*n* = 8) and/or culture (*n* = 7) testing. Seven (77.8%) of these 9 children also had M. tuberculosis-positive Xpert results for their stool samples. In addition, with stool testing, 2 children who were not identified by using Xpert or culture testing of NGA samples were found to have M. tuberculosis-positive Xpert results. In total, by using stool samples, 9 children (7.3%) were identified as having M. tuberculosis. Combining Xpert and/or culture results for NGA specimens and Xpert results for stool specimens, a total of 11 children (8.9%) had bacteriological confirmation (Table 1). Resistance to rifampin was detected for none of the M. tuberculosis-positive children, All 11 children had symptoms suggesting TB, and 8 of them had a TB contact history.

**TABLE 1:** Details of the 11 children for whom M. tuberculosis was detected in the proof-of-principle study in routine settings

Child	Age	Sex	Cough for >2 wk	Fever	Failure to thrive	Reduced playfulness	Contact history	Xpert Ultra result for stool sample (at site)^*a*^	Xpert Ultra result for NGA sample (at site)^*a*^	Solid culture result for NGA sample (at NTRL)	Liquid culture result for NGA sample (at NTRL)
1	Missing	Female	X	X	X	X		MTB detected, medium	MTB detected, high	MTB complex	MTB complex
2	5 yr	Female	X	X	X	X		MTB trace detected	MTB not detected	Negative	Negative
3	3 yr	Male	X		X	X	X	MTB detected, low	MTB not detected	Negative	Negative
4	5 yr	Male	X		X	X	X	MTB detected, medium	MTB detected, high	MTB complex	MTB complex
5	10 yr	Female	X	X	X	X	X	MTB trace detected	MTB detected, low	Negative	MTB complex
6	10 yr	Female	X	X	X	X		MTB trace detected	MTB detected, low	Negative	Negative
7	7 yr	Female	X	X	X	X	X	MTB detected, very low	MTB detected, low	Negative	Negative
8	6 yr	Male	X	X	X	X	X	MTB not detected	MTB trace detected	MTB complex	MTB complex
9	6 mo	Male	X	X	X	X	X	MTB detected, high	MTB detected, high	MTB complex	MTB complex
10	8 mo	Male	X	X	X		X	MTB not detected	MTB detected, very low	Negative	MTB complex
11	4 yr	Female	X	X	X		X	MTB detected, very low	MTB not detected	MTB complex	MTB complex

a These tests were performed in the laboratories at the health care facilities where the children presented. Rifampicin resistance was detected for none of these children. MTB, M. tuberculosis.

Of the 123 Xpert tests conducted on NGA samples, 3 (2.4%) resulted in unsuccessful test results; 2 demonstrated error code 5007 and one had no result. Of the Xpert tests conducted on stool samples, 8 (6.5%) had unsuccessful test results, 3 with error code 2008 and 3 with error code 5007; 1 test was invalid, and 1 had no result. The differences in the proportions of unsuccessful test results for Xpert testing of NGA samples versus testing of stool samples with the SOS method was not statistically significant (*P* = 0.096). Comparing the 83 records with SPC *C_T_* values for both NGA and stool samples indicated that the mean SPC *C_T_* values for the Xpert stool tests were slightly but significantly higher (*P* < 0.01) at 27.9 (95% CI, 27.5 to 28.4 [range, 24.8 to 36.5]), compared to 25.8 (95% CI, 25.5 to 26.0 [range, 23.3 to 31.1]) for NGA samples. All NGA samples were cultured in LJ medium and MGITs. The contamination rate was 6.2% for LJ medium and 8.3% for MGITs. One child had an X-ray available to support the diagnosis (this child is referred to as child 1 in Table 1). Tuberculin skin test results were available for none of the children.

## DISCUSSION

We developed the SOS stool processing method for Xpert testing, which is as simple as sputum processing for Xpert testing, and we demonstrated that this method could detect M. tuberculosis in the stool of children with presumptive TB. In stool samples spiked with M. tuberculosis, the SOS stool processing method showed a similar M. tuberculosis recovery rate and detected higher M. tuberculosis bacterial loads than did the TS method. We also demonstrated that the SOS stool method could be implemented in a routine setting to detect M. tuberculosis in stool samples from children with presumptive PTB at the lower levels of the health care system in an African country. The SOS stool method does not need additional supplies or equipment and can be easily rolled out to every Xpert site, as it requires the same essential materials and equipment as for sputum processing for Xpert testing and, in addition, only a wooden stick for the transfer of stool and pots for collection of stool.

### Method development.

The SPC detects potential inhibition of the PCR by various substances, such as complex polysaccharides, bile salts, and lipids, which are abundant in human stool (19). The more inhibition there is, the higher the SPC *C_T_* value is. As expected, we found that SPC *C_T_* values increased when increasing amounts of stool were used for Xpert testing, which was also observed by Banada and colleagues (12). However, by using the increase of the SPC *C_T_* value from the reference value, we demonstrated that the optimized SOS stool method resulted in a minimal release of PCR inhibitors from the stool. Suboptimal performance of the PCR due to inhibition increases the number of PCR cycles needed to reach the threshold levels of the SPC and moves M. tuberculosis detection closer to the maximum allowed by the proprietary algorithm of Cepheid (*C_T_* of 40). Such high *C_T_* values can lead to invalid results and potentially also to false-negative MTB Xpert results (20). Natural variations in stool appearance and in the presence of PCR inhibitors could influence this balance and require further investigation.

Our study suggests that the additional steps for stool processing used in other processing methods, such as dilution (15), bead beating, and/or filtration (12), are not essential for preparation of the stool samples before Xpert testing. The slightly higher SPC *C_T_* values observed during the method development in phase 1, compared to those during the methods validation in phase 2 of our study, could be explained by the difference in the final proportions of PBS in the supernatant transferred to the cartridge in these experiments, as PBS is known to inhibit the PCR processes (21). Bead beating with glass beads improves homogenization of the stool-SR mixture. However, improved homogenization may lead to formation of a stable suspension of fine debris with low sedimentation rates, complicating the transfer of debris-free supernatant into the Xpert cartridge and increasing the risk of obtaining Xpert error results due to blockage of the microfluid system within the cartridge. The release of inhibitors is also increased when bead beating is used, resulting in elevation of the SPC *C_T_* values. Indeed, in our experiments, as expected, the SPC *C_T_* values were slightly higher when glass beads were added, compared to experiments without beads. However, improved homogenization could also facilitate the release of M. tuberculosis bacilli into the supernatant, thus increasing detection of M. tuberculosis (when present). We could not demonstrate this, as the series of experiments in the first phase of our study were performed with stool specimens from healthy children (without M. tuberculosis bacteria). The aim of filtration of the stool-SR mixture is to remove most of the insoluble particles and PCR inhibitors to prevent clogging of the filters in the Xpert cartridge. Indeed, the SPC *C_T_* values measured in the experiments performed with filtered stool samples were lower than those measured in experiments in which processing methods without filtration were used, suggesting that filtration does remove (some) inhibitors and therefore might improve sensitivity. By removing particles, the rates of tests resulting in an error could potentially be reduced. The Foundation for Innovative New Diagnostics (FIND) developed a prototype of a stool processing kit (SPK), containing reagents and supplies, designed for easier performance of the “Banada method” (11, 12), including the filtration step. The SPK is currently under validation in head-to-head comparison studies in which three centrifuge-free stool processing methods, including our SOS method, are being evaluated.

### Method validation.

The spiked stool experiments, as a prelude to routine diagnostic testing, confirmed that M. tuberculosis could be detected by using the SOS stool method. Although two unsuccessful results were obtained by using the SOS method, the SOS method measured higher M. tuberculosis bacterial loads than the TS method, as was deduced from the semiquantitative Xpert Ultra results. This could be explained by the dilution factor, which is created when stool is transferred into PBS as the second step in the TS method. This dilution step also poses a biosafety risk, because the M. tuberculosis bacteria are not yet inactivated at that stage.

Comparison of our results with the results of the OSF method (14), testing the same stool samples spiked as described previously by Lounnas et al. (14), suggests that the M. tuberculosis recovery rate of the SOS method is slightly better than that of the OSF method. The M. tuberculosis recovery rates for the 29 stool pairs spiked with 10^3^ CFU/g were 93.1% with the SOS stool method (our study) and 70% with the OSF method (14). This might be due to the smaller volume of stool tested and the additional dilution with Sheather’s solution in the OSF method. The rate of unsuccessful tests obtained with the SOS method was lower than that obtained with the OSF method, i.e., 6.9% (our study) and 10% (14), respectively. This observation needs further investigation, and more results on this comparison will be provided by the head-to-head comparison studies mentioned above, in which the OSF method is also included.

### Proof-of-principle study.

The proof-of-principle study confirmed the ability and feasibility of the SOS stool method in combination with Xpert testing to detect M. tuberculosis in stool samples from children with presumptive TB. In total, 9 children with M. tuberculosis were identified by testing stool samples with the SOS method. The high Xpert test success rate of 93.5% shows that the method is robust and easy to perform, as tests were done by multiple laboratory staff members in routine settings in different primary health care facilities in Ethiopia.

Our study showed that the bacterial loads in stool and NGA samples measured by the Xpert assay varied among the children included and ranged from M. tuberculosis trace detected to M. tuberculosis detected, medium. Generally, the bacterial loads measured by Xpert testing of NGA samples were higher than those measured by Xpert testing of stool samples. Indeed, recent meta-analyses (22, 23) suggest that Xpert testing of stool samples (using processing methods other than the SOS stool method) is less sensitive than Xpert testing of NGA or induced sputum samples. The pooled sensitivity and specificity against a microbiological reference standard were 67% (95% CI, 52% to 79%) and 99% (95% CI, 98% to 99%) for stool testing, respectively, according to MacLean et al. (22), and 57% (95% CI, 40% to 72%) and 98% (95% CI, 96% to 99%), respectively, according to Mesman et al. (23).

The difference in Xpert M. tuberculosis-positive results between NGA and stool samples can be explained by the fact that, generally, children have paucibacillary bacterial loads that often are close to the limit of detection of Xpert Ultra testing. Therefore, there is a risk that one sample tests negative while another sample taken from the same child tests positive. Walters and colleagues observed an increase in sensitivity from 44% when only one stool sample was tested with the Xpert assay to 70% when two samples were tested (13).

We observed that, for 2 of 9 children, M. tuberculosis was detected in their stool samples with the Xpert assay, whereas the NGA samples were Xpert and culture negative. In addition, for 1 child, M. tuberculosis was detected with the Xpert assay in the stool sample but not in the NGA sample, although culture results for the NGA sample were M. tuberculosis positive. Detection of M. tuberculosis in stool but not NGA samples could be due to low quantity and/or poor quality of the NGA samples taken or because of a possible intestinal instead of pulmonary etiology of the TB disease (24). Based on the available evidence, stool has been recommended recently to be suitable for a rule-in test (22, 23). However, a negative test result does not *per se* exclude M. tuberculosis, and clinical diagnosis by using well-defined symptoms will remain important in pediatric TB (25). Two children tested negative with stool samples, even after repeat testing, while M. tuberculosis was detected by both Xpert testing and culture of NGA samples. For one of these children, the stool sample was submitted 2 weeks after the NGA sample was obtained and TB treatment had already been started, which could explain the negative stool result. For the other child, the NGA Xpert result was M. tuberculosis trace detected, indicating a very low concentration of M. tuberculosis bacilli close to the limit of detection of the Xpert test.

In our proof-of-principle study, 8 stool samples did not yield a valid Xpert result. For 7 (87.5%) of these, this was potentially related to the nature of the sample and/or the processing method. We retrieved error codes (2008 and 5007) related to technical issues, such as filling of the cartridge chamber with less than 2 ml of supernatant and/or clogged filters. The clogged filters might be a result of leftover debris or lipids in the supernatant transferred to the cartridge. One stool sample yielded no result due to interruption of the Xpert test by a power outage; therefore, this lack of result was related neither to the sample itself nor to the processing of the sample. The single observed invalid result points to too much inhibition, caused by either use of an amount of stool above the recommended 1.0 g or by unknown inhibitors present in that particular stool sample. This underlines the importance of taking the right amount of stool. Whereas more stool increases the chance of detecting M. tuberculosis and thus sensitivity, too much stool may result in a high rate of invalid Xpert results due to release of inhibitors. Thus, training in taking the correct amount of stool needs attention, and the use of bench aids to illustrate the right amount of stool, as well as close supervision during the implementation, is critical. We observed that, over time, staff members became more experienced in the SOS stool method and the rate of unsuccessful Xpert results decreased. Similar to Xpert testing of sputum samples, there is a need to closely monitor the rate of unsuccessful test results and to take corrective actions when the rate exceeds the target (26). The WHO quality indicator target for unsuccessful Xpert results for sputum samples is <5% (27), but there is no such quality indicator for stool samples. Recent publications that applied other stool processing methods also reported >5% unsuccessful tests (13, 14). Furthermore, the mean SPC *C_T_* value for stool samples was higher than for that for NGA samples, which was in line with our expectations because processed stool samples are likely to contain more inhibitors than processed NGA samples. Therefore, there is a need to define specific WHO quality standards for Xpert testing of stool samples.

### Conclusion.

This work shows that a simple and low-cost stool processing method that uses a procedure similar to that used for sputum samples on a large scale worldwide can detect M. tuberculosis and resistance to rifampin at any Xpert site in a routine setting. The method has the potential to enable bacteriological diagnosis of (drug-resistant) TB in children at the lowest levels of health care, directing the start of anti-TB treatment and avoiding lengthy care-seeking pathways and additional costs for the patient and the health care system. More evidence on the diagnostic accuracy and robustness of the SOS stool method is currently being collected.
