# Supplementary material for: The Simple One-Step (SOS) Stool Processing Method for Use with the Xpert MTB/RIF Assay for a Child-Friendly Diagnosis of Tuberculosis Closer to the Point of Care
Source: J Clin Microbiol. 2021 Jul 19;59(8):e00406-21. doi: 10.1128/JCM.00406-21 (PMC8373220; doi:10.1128/JCM.00406-21)
Supplement: Supplemental file 1 — Fig. S1. Download JCM.00406-21-s0001.pdf, PDF file, 140 KB [file jcm.00406-21-s0001.pdf]

Supplemental material Figure S1

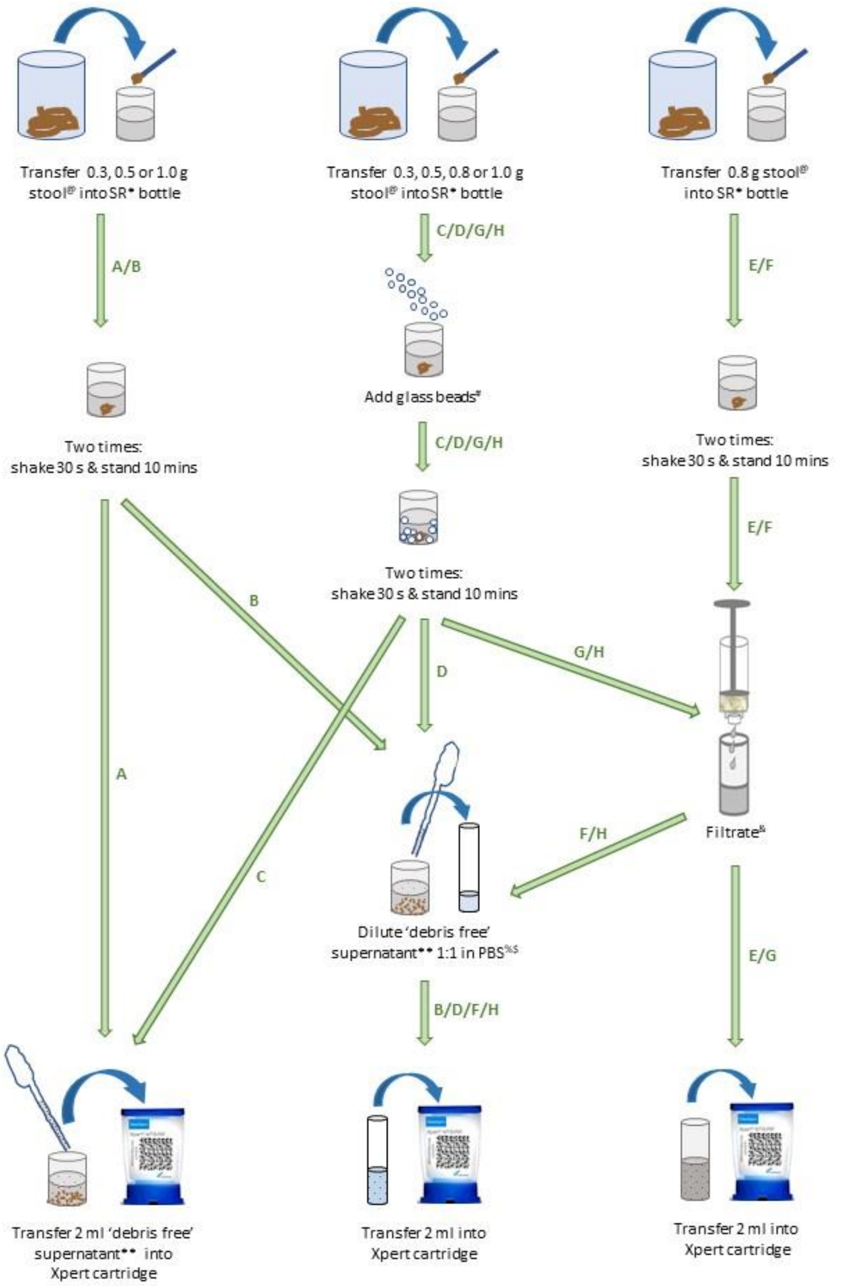

Figure S1. Schematic overview of the first series of laboratory experiments that were used for the SOS stool method protocol development for detection of MTB by using the GeneXpert system. Various amounts of stool from healthy children were processed in different combinations of stool processing steps. In protocol A the proposed SOS stool method was used and the stool was processed similarly to the standard sputum processing for Xpert testing, without additional steps (Bodmer 2012[1], Helb 2010[2]). The other protocols contained additional steps, including dilution (protocol B), bead-beating (protocol C), filtration (protocol E), or combinations of two of these steps (protocols D, F, G) or all three

steps (protocol H). The letters A-H also correspond to the experiments outlined in table S1, in which the amounts of stool tested and the corresponding results are also indicated.

*SR sample reagent (Cepheid); 8 ml mixture of sodium hydroxide (pH>12.5) with isopropanol, provided with every Xpert cartridge.
**After sedimentation by gravitation of the organic debris, carefully -without lifting the bottle and without disturbing the sedimentation- 2 ml of the upper layer of the 'debris free' supernatant was transferred.
[%] PBS, phosphate buffered saline, pH 7.4.
[@] stool was weighed on a precision balance, the stool amount may vary +/- 10%.
[#] +/- 20 glass beads (3.0 mm size) were added to the SR/stool mixture before the first shaking step.
[$] 2 ml of the "debris free" (filtered) supernatant was diluted 1:1 with PBS in a 50 ml tube before transferring it into the Xpert cartridge.
[&] the SR/stool mixture was filtered through a glass wool filter; glass wool was placed at the bottom of a syringe to create +/_5 mm filtration layer after compression with the plunger, where after the prepared syringe was inserted into a 50 ml tube. Filtration was conducted under the gravity condition (not forced with the plunger).

1. Bodmer, T., Ströhle, A. Diagnosing Pulmonary Tuberculosis with the Xpert MTB/RIF Test. J. Vis. Exp. (62), e3547 10.3791/3547, DOI : 10.3791/3547 (2012). http://www.jove.com/video/3547/
2. Helb, D., et al. Rapid Detection of Mycobacterium tuberculosis and Rifampin Resistance by Use of On-Demand, Near-Patient Technology. J. Clin. Microbiol. 48, 229-237, (2010)
