# Supplementary material for: The Simple One-Step (SOS) Stool Processing Method for Use with the Xpert MTB/RIF Assay for a Child-Friendly Diagnosis of Tuberculosis Closer to the Point of Care
Source: J Clin Microbiol. 2021 Jul 19;59(8):e00406-21. doi: 10.1128/JCM.00406-21 (PMC8373220; doi:10.1128/JCM.00406-21)
Supplement: Supplemental file 2 — Table S1. Download JCM.00406-21-s0002.pdf, PDF file, 159 KB [file jcm.00406-21-s0002.pdf]

Supplemental material Table S1

Table S1. Overview of the various experiments conducted during the method development phase of this study and the results of the various protocols conducted for the various amounts of stool tested by Xpert. Results are stratified by experiment and quantity of stool used in each experiment as outlined in figure S1.

Demonstration experiments	Test protocol includes			Amount of stool (gram)							
	Bead-beating	Filtration	Dilution (1:1)	0.3		0.5		0.8		1.0	
				SPC-Ct#	ΔCt‡	SPC-Ct	ΔCt	SPC-Ct	ΔCt	SPC-Ct	ΔCt
A (SOS)	No	No	No	25.5	0.1	29.9	4.5	nd*	nd	36.4	11
B (TS)	No	No	**Yes**	33.3	7.9	33.4	8	nd	nd	33.2	7.8
C	**Yes**	No	No	27.1	1.7	27	1.6	nd	nd	36.6	11.2
D	**Yes**	No	**Yes**	27.4	2	30.2	4.8	nd	nd	Invalid	-
E	No	**Yes**	No	nd	nd	nd	nd	24.4/error	-1	nd	nd
F	No	**Yes**	**Yes**	nd	nd	nd	nd	24.5$	-0.9	nd	nd
G	**Yes**	**Yes**	No	nd	nd	nd	nd	27.0$	1.6	27.6	2.2
H	**Yes**	**Yes**	**Yes**	nd	nd	nd	nd	26.7$	1.3	29.1	3.7

#SPC-Ct; cycle threshold (Ct) of the sample processing control (SPC).
‡ ΔCt was calculated by subtracting the reference (undiluted SR) SPC-Ct value from the SPC-Ct value obtained of each experiment from; The reference SPC-Ct value varied from 24.3 to 26.2 with a mean of 25.4 ± 0.7 over the five runs conducted.
*nd; not done.
$ the results shown are the average of two test runs.
