# Supplementary material for: The Simple One-Step (SOS) Stool Processing Method for Use with the Xpert MTB/RIF Assay for a Child-Friendly Diagnosis of Tuberculosis Closer to the Point of Care
Source: J Clin Microbiol. 2021 Jul 19;59(8):e00406-21. doi: 10.1128/JCM.00406-21 (PMC8373220; doi:10.1128/JCM.00406-21)
Supplement: Supplemental file 3 — Table S2. Download JCM.00406-21-s0003.pdf, PDF file, 78 KB [file jcm.00406-21-s0003.pdf]

Supplemental material

Table S2, Details of the spiked samples and the Xpert Ultra results of the second series of laboratory experiments using the simple one step (SOS) and the two step (TS) in pairs of aliquots from the same sample

Pairs	Stool no.	Stool appearance	Sample processing	Xpert result	SPC -Ct
1	1	semi solid	SOS	MTB trace detected	neg
1	1	semi solid	TS	MTB trace detected	29.4
2	2	soft	SOS	error 2008	neg
2	2	soft	TS	MTB trace detected	neg
3	2	soft	SOS	MTB trace detected	neg
3	2	soft	TS	MTB trace detected	neg
4	3	soft	SOS	MTB detected very low	neg
4	3	soft	TS	MTB detected very low	neg
5	3	soft	SOS	MTB detected very low	neg
5	3	soft	TS	MTB detected very low	neg
6	4	soft	SOS	MTB detected very low	30.2
6	4	soft	TS	MTB not detected	30
7	4	soft	SOS	MTB detected very low	34
7	4	soft	TS	MTB trace detected	28.6
8	5	liquid	SOS	MTB trace detected	neg
8	5	liquid	TS	MTB trace detected	neg
9	5	liquid	SOS	invalid	neg
9	5	liquid	TS	MTB trace detected	neg
10	6	soft	SOS	MTB trace detected	neg
10	6	soft	TS	MTB not detected	26.3
11	6	soft	SOS	MTB trace detected	neg
11	6	soft	TS	MTB detected very low	26.9
12	7	solid	SOS	MTB detected very low	28.8
12	7	solid	TS	MTB detected very low	28.5
13	7	solid	SOS	MTB detected very low	31.3
13	7	solid	TS	MTB trace detected	26.8
14	8	semi liquid	SOS	MTB trace detected	neg
14	8	semi liquid	TS	MTB trace detected	neg
15	8	semi liquid	SOS	MTB trace detected	neg
15	8	semi liquid	TS	MTB trace detected	neg
16	9	soft	SOS	MTB trace detected	neg
16	9	soft	TS	MTB trace detected	36.9
17	9	soft	SOS	MTB detected very low	38.6
17	9	soft	TS	MTB trace detected	29.1
18	10	soft	SOS	MTB detected low	36.2
18	10	soft	TS	MTB detected low	29.2
19	10	soft	SOS	MTB detected low	33.6
19	10	soft	TS	MTB detected very low	neg

20	11	semi liquid	SOS	MTB detected very low	32.7
20	11	semi liquid	TS	MTB trace detected	27.8
21	11	semi liquid	SOS	MTB detected low	30.4
21	11	semi liquid	TS	MTB trace detected	neg
22	12	soft	SOS	MTB detected low	35.7
22	12	soft	TS	MTB detected very low	26.6
23	12	soft	SOS	MTB detected medium	27.9
23	12	soft	TS	MTB detected low	27
24	13	soft	SOS	MTB detected very low	neg
24	13	soft	TS	MTB detected very low	36.9
25	13	soft	SOS	MTB detected very low	33.7
25	13	soft	TS	MTB detected very low	37
26	14	soft	SOS	MTB detected very low	neg
26	14	soft	TS	MTB trace detected	29.2
27	14	soft	SOS	MTB detected very low	38.1
27	14	soft	TS	MTB trace detected	26.4
28	15	liquid	SOS	MTB detected medium	32.2
28	15	liquid	TS	MTB detected very low	29.6
29	15	liquid	SOS	MTB detected medium	31.8
29	15	liquid	TS	MTB detected very low	26.9

* SOS, Simple one step stool method, **TS two step stool method, @neg, negative SPC-Ct value
